# The Bezold-Jarisch Reflex and The Inflammatory Response Modulation in Unanesthetized Endotoxemic Rats

**DOI:** 10.3389/fphys.2021.745285

**Published:** 2021-09-20

**Authors:** Fernanda Brognara, Jaci Airton Castania, Aline Barbosa Ribeiro, Nilton Nascimento Santos-Júnior, Helio Cesar Salgado

**Affiliations:** ^1^Department of Physiology, Ribeirão Preto Medical School, University of São Paulo, Ribeirão Preto, Brazil; ^2^Minas Gerais State University, Passos, Brazil

**Keywords:** Bezold-Jarisch reflex, parasympathetic activation, sympathetic inhibition, phenylbiguanide, inflammation, rats

## Abstract

Evidence indicates that the activation of the parasympathetic branch of the autonomic nervous system may be effective in treating inflammatory diseases. Previously, we have described that baroreflex activation displays anti-inflammatory properties. Analogous to the baroreflex, the Bezold-Jarisch reflex also promotes parasympathetic activation with simultaneous inhibition of the sympathetic system. Thus, the present study aimed to evaluate whether the activation of the Bezold-Jarisch reflex would also have the ability to reduce inflammation in unanesthetized rats. We used lipopolysaccharide (LPS) injection (5mg/kg, i.p.) to induce systemic inflammation in male Wistar Hannover rats and phenylbiguanide (PBG) administration (5μg/kg, i.v.) to activate the Bezold-Jarisch reflex. Spleen, heart, hypothalamus, and blood samples were collected to determine the levels of cytokines. Compared to baseline, PBG reduced the arterial pressure (115±2 vs. 88±5mmHg) and heart rate (380±7 vs. 114±26bpm), immediately after its administration, confirming the activation of the parasympathetic system and inhibition of the sympathetic system. From the immunological point of view, the activation of the Bezold-Jarisch reflex decreased the plasma levels of TNF (LPS: 775±209 vs. PBG + LPS: 248±30pg/ml) and IL-6 levels in the spleen (LPS: 39±6 vs. PBG + LPS: 24±4pg/mg of tissue). However, it did not change the other cytokines in the plasma or the other tissues evaluated. These findings confirm that the activation of the Bezold-Jarisch reflex can modulate inflammation and support the understanding that the cardiovascular reflexes regulate the immune system.

## Introduction

Several studies highlighted the role of the parasympathetic system in controlling the inflammation in numerous inflammatory diseases (Pavlov et al., 2020). More recently, the baroreflex was identified as a modulator of the inflammatory response (Bassi et al., 2015; Brognara et al., 2018), in addition to its typical function of regulating arterial pressure (Krieger et al., 1982; Chapleau et al., 1988). When activated, the baroreflex results in a neural response that comprises parasympathetic activation and sympathetic activity inhibition contributing to arterial pressure homeostasis (Krieger et al., 1982; Chapleau et al., 1988). Thus, the anti-inflammatory role played by baroreflex activation is probably associated with its influence on both branches – sympathetic and parasympathetic – of the autonomic nervous system.

The Bezold-Jarisch reflex is a cardiopulmonary reflex in which the parasympathetic innervation of the cardiopulmonary region is crucial to regulate heart rate, systemic vasomotor tone, and respiration, and when activated, promotes bradycardia, hypotension, and apnea (Aviado and Guevara Aviado, 2001). In experimental studies, the Bezold-Jarisch reflex is activated by some pharmacological agents, including a selective serotonergic 5-HT3 receptor agonist, the phenylbiguanide (PBG; Daly and Kirkman, 1988; Bell et al., 1993; Salo et al., 2007; Bezerra et al., 2011; Silva et al., 2016). Analogous to the baroreflex, the Bezold-Jarisch reflex also promotes parasympathetic activation with concomitant inhibition of the sympathetic nervous system. In addition, the baroreflex and the Bezold-Jarisch reflex share a common central pathway in the brainstem (Verberne and Guyenet, 1992; Verberne et al., 1999; Kashihara, 2009).

Considering that baroreflex activation has anti-inflammatory properties, it is reasonable to expect that the Bezold-Jarisch reflex would also have a similar ability since both reflexes share the same central pathway and analogous efferent autonomic response. Therefore, this study aimed to evaluate if the activation of the Bezold-Jarisch reflex can modulate the systemic inflammatory response in unanesthetized endotoxemic rats.

## Materials and Methods

### Experimental Animals

Male Wistar Hannover rats (250–300g) from the Main Animal Facility of the University of São Paulo (Campus of Ribeirão Preto; Ribeirão Preto, SP, Brazil) were used to perform the study. The animals were kept under a constant light-dark cycle (12h) and controlled temperature (23°C) with free access to water and food. The experimental protocol of the present study was evaluated and approved by the Committee of Ethics in Animal Research from the Ribeirão Preto Medical School – University of São Paulo (protocol #161/2016).

### Surgical Procedures

A cocktail of ketamine and xylazine (50mg/kg and 10mg/kg, i.p.) was used to anesthetize the animals to perform the surgical procedures of catheterization. The left femoral artery and vein were catheterized with polyethylene tubing (PE-50 soldered to a PE-10 polyethylene tube; Intramedic, Clay Adams, Parsippany, NJ, United States) for arterial pressure recording and PBG or saline (vehicle) administration, respectively. Furthermore, a catheter (PE-50 polyethylene tube; Intramedic, Clay Adams, Parsippany, NJ, United States) was inserted into the abdominal cavity for the administration of (lipopolysaccharide) LPS from *Escherichia coli* 0111:B4 (Sigma-Aldrich, St. Louis, MO, United States) or saline (vehicle). The catheters were pulled up through a subcutaneous track to the animal’s neck and exteriorized at the nape, and surgical incisions were sutured. Analgesic (tramadol hydrochloride: 2mg/kg, s.c.) was administered to avoid pain.

### Experimental Protocol

Forty-eight hours after the surgical procedures, the rats were assigned into four groups:

1. **Sal+Sal:** saline administration (i.v.) followed by saline injection (i.p.).
2. **PBG+Sal:** phenylbiguanide administration (i.v.) followed by saline injection (i.p.).
3. **Sal+LPS:** saline administration (i.v.) followed by LPS injection (i.p.).
4. **PBG+LPS:** phenylbiguanide administration (i.v.) followed by LPS injection (i.p.).

With the animal unanesthetized, the arterial catheter was connected to a polyethylene tube, filled with saline solution, hanging loosely over the cage. This extension was connected to the pressure transducer attached to the recording system. The venous and peritoneal catheters were connected to other polyethylene tubes in order to allow for drug administration. Although the animals were still connected to extensor tubes, the length of extensions allowed the animals to move freely throughout the recording period. Thus, the experiment was conducted with the animals moving freely in their cages, but carefully supervised by the investigator. Silence was maintained in the room to minimize environmental stress. The experimental protocol consisted of 30min of basal recordings of pulsatile arterial pressure, followed by the administration of PBG (5μg/kg, i.v.; Bezerra et al., 2011) – to promote the activation of the Bezold-Jarisch reflex – or saline (as control). Five minutes later, the LPS (5mg/kg, i.p.; Brognara et al., 2018) was administered – to induce systemic inflammation – or saline (as control) was injected. The arterial pressure was recorded until completing 90min after LPS or saline injection. Next, the arterial pressure recording was suspended, and a blood sample was collected through the catheter from the femoral artery with heparin. Then, the rats were killed by decapitation for quick collection of samples from the spleen, the heart, and the whole hypothalamus, which were immediately frozen in liquid nitrogen. Blood samples were kept on ice until centrifugation at 3500rpm for 15min at 4°C. The plasma was then collected, and all biological material was stored at −80°C.

### Arterial Pressure Recording and Analysis

The pulsatile arterial pressure was recorded using a pressure transducer (MLT844; ADInstruments, Bella Vista, Australia), an amplifier (ML224; ADInstruments, Bella Vista, Australia), and an analog-to-digital interface (PowerLab, ADInstruments, Bella Vista, Australia). The mean arterial pressure and heart rate analysis were carried out using computer software (LabChart 7.0, ADInstruments, Bella Vista, Australia). The analysis from the prompt response to saline or PBG administration was carried out within the first 5s after its injection. For the other moments, a period of 10min was used.

### Cytokine Measurements

On the day of the assay, the tissue samples were homogenized in 0.5ml of PBS using a homogenizer (Polytron-PT-3100, Evisa, Kinematica, Luzern, Switzerland) and then centrifuged at 1000rpm for 10min at 4°C. The plasma and tissue supernatant samples were used to measure the cytokine [tumor necrosis factor-alpha (TNF-α), interleukin (IL)-6, IL-1β, and IL-10] levels by the immunoenzymatic method (ELISA) using Duo set kits from R&D Systems (Minneapolis, MN, United States) according to the manufacturer’s instructions.

### Statistical Analysis

The analysis of the hemodynamic data was carried out using two-way ANOVA for repeated measures, followed by the Tukey *post-hoc* test. The analysis of inflammatory mediators was carried out using one-way ANOVA, followed by the same *post-hoc* test. Differences were considered statistically significant if *p*<0.05. Statistical analysis was performed using the SigmaPlot 12.0 software (Systat Software, San Jose, CA, United States), and the results are presented as mean ± standard error of the mean.

## Results

### Hemodynamic Response to Phenylbiguanide

**Figure 1:**
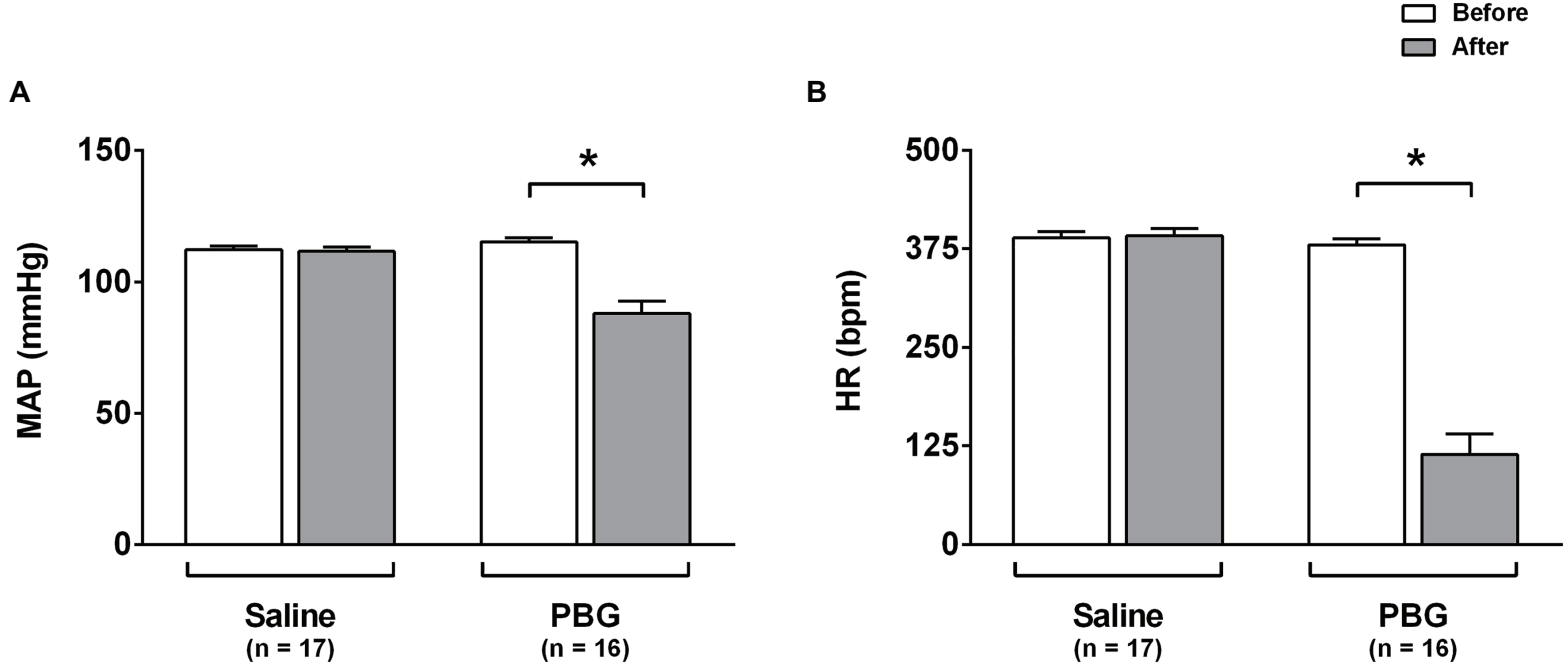
Hemodynamic response to phenylbiguanide. Bar graphs show the mean arterial pressure [MAP **(A)**] and heart rate [HR **(B)**] at baseline (white bars) and after administration (gray bars) of phenylbiguanide (PBG) or saline. LPS, lipopolysaccharide; PBG, phenylbiguanide; and Sal, saline. ^*^p<0.05. Two-way ANOVA for repeated measures with Tukey’s *post-hoc* test.

Right after saline administration (first 5s), no change was seen in the arterial pressure (112±1 vs. 112±2mmHg; *p*=0.847) or heart rate (389±8 vs. 392±9bpm; *p=* 0.887; Figures 1A,B). However, the prompt response observed after PBG administration was characterized by a reduction in arterial pressure (115±2 vs. 88±5mmHg; *p*<0.001; Figure 1A) and an abrupt bradycardia (380±7 vs. 114±26bpm; *p*<0.001; Figure 1B).

### Time Course of Arterial Pressure and Heart Rate

**Figure 2:**
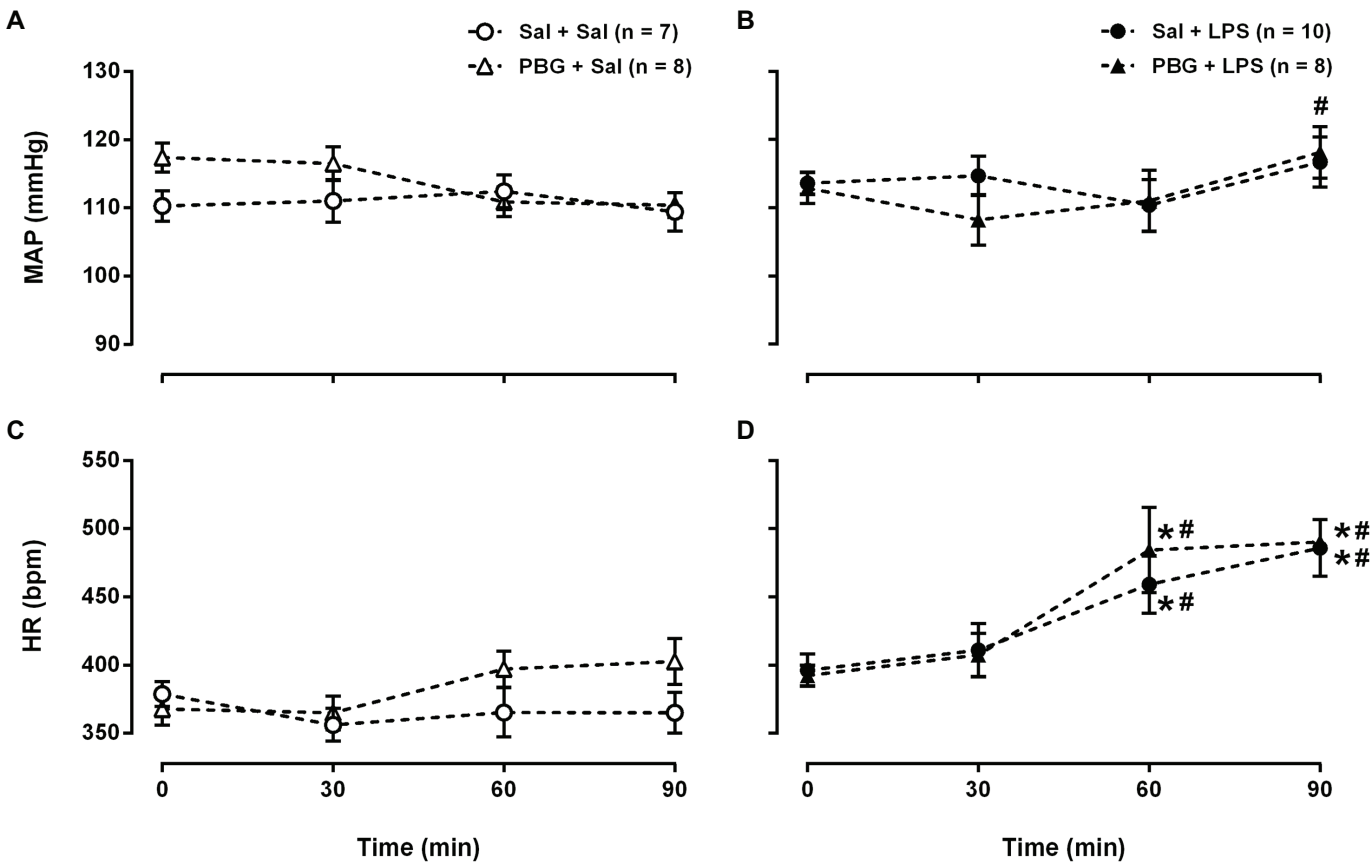
Time course of hemodynamic response to phenylbiguanide and LPS. Mean arterial pressure [MAP **(A)** and **(B)**] and heart rate [HR **(C)** and **(D)**] at baseline (time zero), 30, 60, and 90min after the administration of LPS or saline from the different groups. LPS, lipopolysaccharide; PBG, phenylbiguanide; and Sal, saline. ^*^p<0.05 vs. 0min; ^#^*p*<0.05 vs. 30min. Two-way ANOVA for repeated measures with Tukey’s *post-hoc* test.

Comparing all the groups at each moment, no changes in arterial pressure were observed over time at any time evaluated (all *p*>0.05; Figures 2A,B). That is to say, the injection of LPS or PBG did not promote alterations in arterial pressure over 90min. Nevertheless, comparing all the moments within the same group, there is an increase in arterial pressure in the PBG+LPS group at 90min compared to 30min (108±4 vs. 118±4mmHg; *p*=0.008; Figure 2B). Saline or PBG administration did not change the heart rate over time (Figure 2C). However, all groups that received LPS had an increase in the heart rate starting 60min after LPS injection which was maintained until the end of the protocol at 90min (Figure 2D). In addition, PBG administration did not prevent the tachycardia induced by LPS over time (Figure 2D).

### Bezold-Jarisch Reflex Activation and Cytokine Level

**Figure 3:**
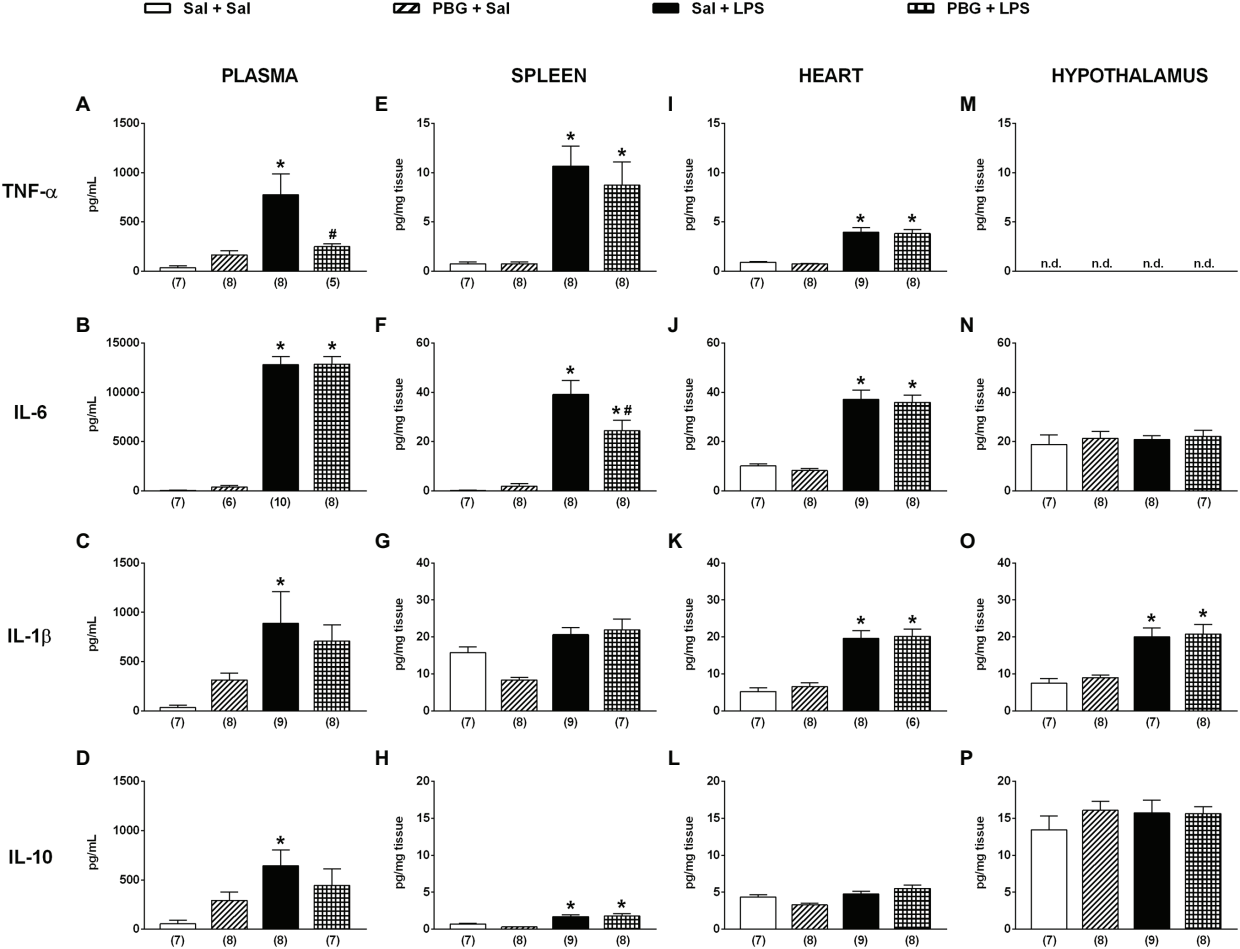
Bezold-Jarisch reflex activation decreased the release of TNF-α induced by LPS in plasma and IL-6 in the spleen. Bar graphs show the levels of TNF-α, IL-6, IL-1β, and IL-10 in plasma **(A-D)**, spleen **(E-H)**, heart **(I-L)**, and hypothalamus **(M-P)** 90min after the administration of LPS or saline in the different groups. IL-6, interleukin 6; IL-1β, interleukin 1β; IL-10, interleukin 10; LPS, lipopolysaccharide; n.d, not detected; PBG, phenylbiguanide; Sal, saline; and TNF-α, tumor necrosis factor-alpha. ^*^p<0.05 vs. Sal + Sal group; ^#^*p*<0.05 vs. Sal + LPS group. One-way ANOVA with Tukey’s *post-hoc* test. The number in parentheses represents the number of samples of each group.

The administration of saline associated, or not, with PBG did not modify the plasma cytokine levels (Figures 3A–D). Instead, the administration of LPS increased all the cytokines evaluated in plasma 90min after its injection (all *p*<0.05; Figures 3A–D). The activation of the Bezold-Jarisch reflex decreased, by more than 60%, the release of TNF-α in plasma 90min after LPS compared to Sal + LPS (Sal + LPS: 775±209 vs. PBG + LPS: 248±30pg/ml; *p*=0.044; Figure 3A). However, it did not prevent the increase of the other cytokines induced by LPS (Figures 3B–D). The analysis of tissue cytokines showed that LPS administration augmented most of the cytokines analyzed in the spleen (Figures 3E–H), heart (Figures 3I–L), and hypothalamus (Figures 3N–P). In the spleen, the activation of the Bezold-Jarisch reflex reduced the IL-6 release induced by LPS (Sal + LPS: 39±6 vs. PBG + LPS: 24±4pg/mg of tissue; *p*=0.037; Figure 3F) but did not decrease the levels of the other cytokines induced by LPS (Figures 3E,G,H). In the heart and hypothalamus, the administration of PBG, associated with LPS, did not reduce the release of any cytokine evaluated in the present study (Figures 3I–P). Undetected levels of TNF-α were observed in the hypothalamus (Figure 3M).

## Discussion

Classically, the activation of the Bezold-Jarisch reflex promotes hypotension and bradycardia (Aviado and Guevara Aviado, 2001). The reflex vasodilation of the Bezold-Jarisch reflex is, in fact, because of the inhibition of the sympathetic vasoconstrictor tone (Bell et al., 1993; Verberne et al., 2003). This sympathetic inhibition due to the activation of the Bezold-Jarisch reflex was demonstrated in the lumbar (Verberne and Guyenet, 1992; Sartor and Verberne, 2006), splanchnic (Sartor and Verberne, 2006), splenic (Weaver et al., 1984), and renal nerves (Weaver et al., 1984; Bell et al., 1993). On the other hand, the bradycardia in the Bezold-Jarisch reflex is predominantly due to parasympathetic activation (Daly and Kirkman, 1988; Verberne and Guyenet, 1992). Of note, the hypotension and bradycardia promoted by the activation of the Bezold-Jarisch reflex, evoked by PBG administration, were observed in both anesthetized (Salo et al., 2007; Futuro Neto et al., 2011) and unanesthetized (Chianca and Machado, 1996; Bezerra et al., 2011; Silva et al., 2016) rats. Both responses were observed in the present study indicating that the administration of PBG promoted conspicuous parasympathetic activation and sympathetic inhibition, validating the activation of the Bezold-Jarisch reflex in the chosen model.

The tachycardia observed after LPS administration in the present study aligns with the literature (Altavilla et al., 2002; Brognara et al., 2019). Regarding the arterial pressure response, some studies reported that, over time, LPS reduced the arterial pressure (Mehanna et al., 2007; Sallam et al., 2018). On the other hand, other reports described an increase (Lee et al., 2005; Brognara et al., 2019), or even no changes in the arterial pressure (Brognara et al., 2018) due to LPS administration in unanesthetized rats. Thus, the effects of LPS in the arterial pressure are quite variable in rats, and definitely involve many factors, such as gender, strain, quality and dose of the LPS, and level of anesthesia, among others. The increase in the peripheral vascular resistance over time after LPS administration, due to a rise in sympathetic nerve activity observed in anesthetized rats (Tohyama et al., 2018) and the augmented sympathetic modulation of the vessels demonstrated by the power of the low frequency band of the systolic arterial pressure in unanesthetized rats (Brognara et al., 2019), has also been proposed as a mechanism to explain the LPS impact on arterial pressure.

The central activation pathways for the Bezold-Jarisch reflex consist of the same paths as the baroreflex (Verberne and Guyenet, 1992; Verberne et al., 1999; Kashihara, 2009). Considering that previous reports from our laboratory demonstrated that the stimulation of the baroreflex promoted anti-inflammatory effects in rats (Bassi et al., 2015; Brognara et al., 2018), it was expected that the activation of the Bezold-Jarisch reflex would also produce the same result. Our findings showed that the activation of the Bezold-Jarisch reflex reduced the cytokine release induced by systemic inflammation in unanesthetized rats. However, the anti-inflammatory effects of the activation of the Bezold-Jarisch reflex were not the same as those shown for the baroreflex activation. Brognara et al. (2018) demonstrated that baroreflex activation reduced TNF, IL-6, IL1β, and IL-10 in the hypothalamus of endotoxemic rats without changing the levels of the same cytokines in plasma, spleen, or heart. In the current study, the Bezold-Jarisch reflex did not decrease the inflammation in the hypothalamus or the heart but reduced the levels of TNF-α in the plasma and IL-6 in the spleen. These data indicate that as: (1) although limited to some cytokines and territories, the Bezold-Jarisch reflex exhibited systemic anti-inflammatory modulation and (2) that parasympathetic activation and sympathetic inhibition promoted by the Bezold-Jarisch reflex have different anti-inflammatory properties than those resulting from baroreflex stimulation.

It is noteworthy that the study by Brognara et al. (2018) was also conducted on unanesthetized rats which were subjected to the induction of systemic inflammation through the administration of LPS, as in the present study. However, an essential factor may justify the differences observed in the anti-inflammatory effects promoted by each reflex: The method chosen to activate the parasympathetic and inhibit the sympathetic nervous system. In the experiments from Brognara et al. (2018), the baroreflex was activated through electrical stimulation of the aortic depressor nerve; in other words, a neurostimulation approach was used. In the current study, a pharmacological method was chosen to trigger the Bezold-Jarisch reflex. Thus, although the efferent autonomic response was practically the same for both reflexes, resulting in parasympathetic activation and concomitant sympathetic inhibition, the different afferent approaches used to activate these reflexes appeared to interfere with the effector inflammatory response.

In the present study, the anti-inflammatory effect of the Bezold-Jarisch reflex was limited for TNF-α in plasma and IL-6 in the spleen. Nevertheless, although limited to some cytokines, these data deserve special attention since this reduction by more than 60% in circulating TNF-α could produce a crucial biological role, as TNF-α alone can induce a local and systemic inflammatory profile (Tracey et al., 1986, 1987). Likewise, the cytokine reduction in the spleen is consistent with “cholinergic anti-inflammatory pathway” activation (Tracey, 2002). It is possible that efferent parasympathetic pathway – stimulated by the Bezold-Jarisch reflex – has activated, *via* the vagus nerve and the splenic nerves, which in turn resulted in the recruitment of acetylcholine-producing T cells in the spleen and downregulated the inflammation by the alpha-7 nicotinic acetylcholine receptor on macrophages, as previously proposed in the literature on the “cholinergic anti-inflammatory pathway” (Wang et al., 2003; Tracey, 2010; Rosas-Ballina et al., 2011; Dantzer, 2018). Thus, this reduction of TNF in plasma and IL-6 in the spleen suggests that the activation of the Bezold-Jarisch reflex can have an even more significant biological function than previously believed, involving an anti-inflammatory role. However, further studies are needed to identify whether these anti-inflammatory effects promoted by the activation of the Bezold-Jarisch reflex are predominantly due to parasympathetic activation or sympathetic inhibition.

Of note, the influence of the Bezold-Jarisch reflex on the other cytokines in plasma (IL-6, IL-1β, and IL-10) and the spleen (TNF-α, IL-1β, and IL-10) may have a different time course, occurring later, or even earlier. That is to say, the anti-inflammatory effect produced by the parasympathetic activation and sympathetic inhibition due to the activation of the Bezold-Jarisch reflex would require more, or less, than 90min after LPS to be effective. In that regard, the literature has suggested that the anti-inflammatory effects promoted by vagus nerve stimulation are related to how long after vagal activation the injury is induced, suggesting that this anti-inflammatory pathway needs a complex neuroimmune interaction that may not occur immediately (Inoue et al., 2016). Therefore, we comprehend that the data presented here are preliminary and need further studies to deeply understand the role of the Bezold-Jarisch reflex in controlling inflammation. The conceivable studies would involve the evaluation of the cytokine levels in plasma and tissues at different times during the development of the systemic inflammatory response in unanesthetized rats; a longer period for the activation of the Bezold-Jarisch reflex before inducing the systemic inflammation; tests of different doses of LPS and PBG; and also subdiaphragmatic vagotomy to exclude a non-neuronal effect.

Recently, it was demonstrated that the magnitude of the bradycardic response promoted by the activation of the Bezold-Jarisch reflex is increased 3 and 24h after LPS administration in rats (Amorim et al., 2019). The authors suggest that during systemic inflammation, the increase in the cardiac component of the Bezold-Jarisch reflex could be beneficial to modulate the inflammatory response. Although Amorim et al. (2019) evaluated the Bezold-Jarisch reflex after LPS administration and, in the present study, the activation of this reflex was conducted before the induction of the immune challenge, the current data confirm the influence of the activation of the Bezold-Jarisch reflex on the modulation of the systemic inflammation in rats, in agreement with the literature speculated previously.

In recent years, serotonin has been associated with several functions of the immune system in addition to its role in regulating other physiological mechanisms (Herr et al., 2017). Immune cells express serotonin receptors, including the 5-HT3 receptor (Khan and Poisson, 1999), modulating the inflammatory response. Serotonin reduced cytokine production in human monocytes (Arzt et al., 1991; Cloëz-Tayarani et al., 2003), in primary rat aortic smooth muscle cells (Yu et al., 2008), and also *in vivo* (Nau et al., 2013). Moreover, central serotonin administration decreased plasma and spleen cytokine release induced by LPS in rats (Mota et al., 2019). Serotonin also has a pro-inflammatory effect stimulating the release of cytokine (Ito et al., 2000). This dual role of serotonin may be related to the serotonin receptor subtype class that is activated (Dürk et al., 2005). Selective activation of serotonin 5-HT2A receptors prevented the IL-6 release during a systemic inflammatory response (Nau et al., 2013). On the other hand, the inhibition of 5-HT3 receptors abolished the release of LPS-induced cytokine, suggesting that this serotonin receptor subtype activation participates in the release of pro-inflammatory cytokines, possessing not an anti-inflammatory but a pro-inflammatory function (Fiebich et al., 2004). Thus, since a selective serotonergic 5-HT3 receptor agonist was used to activate the Bezold-Jarisch reflex, it is reasonable to suggest that the anti-inflammatory effects observed in the present study are due to the Bezold-Jarisch reflex activation and not to the direct effect of serotonin receptor stimulation in the immune cells.

In conclusion, this is the first study confirming that the activation of the Bezold-Jarisch reflex induced by PBG administration possesses specific anti-inflammatory effects in rats. In other words, PBG administration to unanesthetized endotoxemic rats elicits the activation of the Bezold-Jarisch reflex and can modulate the release of TNF-α in plasma and IL-6 in the spleen. This novel mechanism of neuromodulation will undoubtedly present new noteworthy questions concerning the investigation of the neural control of inflammation. Further studies should be conducted under different inflammatory conditions to deeply understand the role of cardiovascular reflexes in the inflammatory response, including the mechanisms involved in the activation of the Bezold-Jarisch reflex and the reduction in production and release of cytokines.

## Data Availability Statement

The raw data supporting the conclusions of this article will be made available by the authors, without undue reservation.

## Ethics Statement

The animal study was reviewed and approved by the Committee of Ethics in Animal Research from the Ribeirão Preto Medical School – University of São Paulo (protocol # 161/2016).

## Author Contributions

FB and HS: conception or design of the work. FB, JC, AR, and NS-J: acquisition or analysis or interpretation of data for the work. FB, JC, AR, NS-J, and HS: drafting the work or revising it critically for important intellectual content. All authors approved the final version of the manuscript and agreed to be accountable for all aspects of the work in ensuring that questions related to the accuracy or integrity of any part of the work are appropriately investigated and resolved.

## Funding

This work was supported by the São Paulo Research Foundation (FAPESP; process #2013/20549–7; #2017/05163–6; #2018/20939–3; #2018/10455–9; and #2020/06043–7), Foundation for the Support of Teaching, Research and Service (FAEPA; process #580/21 and #581/21), and the Academic Excellence Program (PROEX) from the Coordination for the Improvement of Higher Education Personnel (CAPES; process #88887.505419/2020–00).

## Conflict of Interest

The authors declare that the research was conducted in the absence of any commercial or financial relationships that could be construed as a potential conflict of interest.

## Publisher’s Note

All claims expressed in this article are solely those of the authors and do not necessarily represent those of their affiliated organizations, or those of the publisher, the editors and the reviewers. Any product that may be evaluated in this article, or claim that may be made by its manufacturer, is not guaranteed or endorsed by the publisher.
